# Commentary: Breaking tradition: should biostatistics doctoral qualifying exams evolve to better serve our students' ability to demonstrate readiness to conduct independent research?

**DOI:** 10.3389/fpubh.2025.1658570

**Published:** 2025-09-12

**Authors:** Andrew J. Spieker

**Affiliations:** Vanderbilt University Medical Center, Nashville, TN, United States

**Keywords:** biostatistics, curriculum, evaluation, qualifying exam, standards

## Introduction

I commend Drs. Bellamy and Sullivan for initiating an important conversation about the role of qualifying examinations (QEs) in biostatistics graduate programs, and echo their sentiments regarding the importance of fostering an inclusive and welcoming environment for trainees (1). Bellamy and Sullivan conclude their report with a call to action: “…[*to*] *become clear on the purpose of the QE, beyond being an arbitrary milestone along the doctoral training continuum*.” They provide compelling support for their case that contemporary graduate training programs have room for growth in this respect. The major purpose of my commentary is to take specific steps toward addressing this.

## Characterizing the role of the QEs

Since 2021, I have led the writing/evaluation of the QEs for Vanderbilt University's graduate program in biostatistics. For me, the timing of this discussion is fortuitous and opportune as I transition into the role of Director of Graduate Studies. Our program's QEs are a mixture of in-class and take-home problems oriented toward theory, methods, and application. At the time of this writing, our program's student handbook asserts the following:

*The primary role of the comprehensive examinations is to aid the graduate faculty in assessing whether students are sufficiently prepared for the next stage of their graduate training, including advanced coursework and development of a thesis or dissertation*.

In some respects, this parallels what Bellamy and Sullivan articulate (and challenge) as a commonly purported argument in favor of QEs. I view the above statement of purpose as adequate in distinguishing a QE from an arbitrary milestone. On the other hand, Bellamy and Sullivan raise a legitimate question as to the optimality of the QE in achieving this goal. I believe optimality hinges upon the care put into developing the examinations and the program's sustained communication with its students. Below, I offer recommendations that balance fairness with the high standards that our students expect us to maintain.

1. Be transparent, forthcoming, and available. For several years, I have held sessions to explain the QE process in detail as early as six months prior. When providing tips, I've emphasized the balance between individual- and group-study. I've pointed students to the website that hosts several previous exams (publicly available and easy to access) so they can acclimate to the exam format. Finally, I've offered review sessions each year, and each year (to my delight) students have taken me up on this.
2. Ensure balance in scope and difficulty. Many programs delegate exam writing across graduate faculty to ensure the burden (considerable as it is) is not unduly placed on any one person. Without some degree of centralized oversight, however, this runs the risk of an imbalanced or otherwise unreasonable examination. I share Bellamy and Sullivan's stance on the counterproductivity of questions designed to be difficult for difficulty's sake. I have insisted not only that each question is vetted by the instructor of the course corresponding to that question's topic, but that each examination is vetted in its entirety by at least one other faculty member for scope, clarity, and alignment.
3. Mind the modernity. As our expectations of proficiency in certain topics (e.g., computing) increase over time, the direct applicability of some historically emphasized topics in mathematical statistics wane. While it requires some creativity in the exam writing process, we can reflect these changes by periodically updating the exam's emphasis.
4. Encourage students to seek resources for accommodations. We can be sensitive to heterogeneity in learning styles. I encourage students who believe they may be eligible for accommodations to consult with the student support center on campus. Many times, students who may be eligible for accommodations may not be aware of this important service until it is brought to their attention.
5. Use the QE as an aid. Our program's current characterization of the QE clarifies that it is not considered in isolation. As a medium-sized program, we have the luxury of being able to take an individualized approach to remediation (e.g., re-doing a problem in a time-limited open-book setting, retaking a course, completing a follow-up oral examination, or serving as a TA for a targeted course) following holistic discussions of a student's progress (with the QE treated as a piece—rather than the whole—of the puzzle). To the extent possible, decisions should be based on a pattern of signals.

The above is not an exhaustive characterization of the measures our program has taken to ensure the quality of our QEs, but is instead a sampling of the sorts of things I believe may alleviate some concerns put forth by Bellamy and Sullivan. Fundamentally, I view the QEs as a motivation for students to consolidate their understanding. I also view proficiency in the foundations of mathematical statistics as a prerequisite to many contemporary advanced topics; nevertheless, we can certainly acknowledge the importance of the foundations without endorsing the deeply disturbing viewpoints of some of their developers. I further posit that well-constructed QEs have the following potential advantages toward the goal of inclusivity:

1. Though a QE may be imperfect, we must consider the unintended consequences of removing an objective indicator of performance from consideration. In so doing, we could inadvertently increase reliance on the unconscious biases we seek to avoid.
2. Students taking a longer time to process and consolidate material may benefit immensely from having dedicated time to study, followed by an additional chance to demonstrate what they have learned. This “glass-half-full” perspective on the utility of a QE is in direct opposition to the more pessimistic perspective of a QE as a gatekeeping tactic. In fairness to the perspective of Bellamy and Sullivan, this point is only relevant insofar as the QE actually reflects material a student can be reasonably expected to master.
3. A QE can be a useful tool for identifying a student's gaps. Again, this point may only be relevant to the degree that we communicate these gaps. Some programs, including our own, have historically resisted providing detailed feedback on these examinations—although we have begun to reconsider this approach in recent years. It may be time to reevaluate our historical choice to withhold feedback to our students, or at least better justify this choice.

## Discussion

My perspective is shaped by my longstanding attempts to navigate the tension between rigorous standards and strong advocacy for our students. Nevertheless, the scope of my response is limited to evaluation of a program's existing students and does not address the challenges we face surrounding representation (i.e., issues more closely tied to recruitment, an aspect of graduate training I have not led and therefore delegate to those with more direct experience for their commentary). While some aspects of my perspective contrast with those of Bellamy and Sullivan, I suspect we agree on the importance of thoughtful implementation of non-arbitrary evaluative procedures.
